# Supplementary material for: Cryo-EM captures early ribosome assembly in action
Source: Nat Commun. 2023 Feb 17;14:898. doi: 10.1038/s41467-023-36607-9 (PMC9935924; doi:10.1038/s41467-023-36607-9)
Supplement: Supplementary file 1 — Supplementary Information [file 41467_2023_36607_MOESM1_ESM.docx]

**Cryo-EM captures early ribosome assembly in action**

Bo Qin^1^†, Simon M. Lauer^1^†, Annika Balke^2^, Carlos H. Vieira-Vieira^3,4^, Jörg Bürger^1,5^, Thorsten Mielke^5^, Matthias Selbach^3,6^, Patrick Scheerer^2^, Christian M. T. Spahn^1^* and Rainer Nikolay^1,7^*

*Corresponding author. Emails: [christian.spahn@charite.de](mailto:christian.spahn@charite.de), [nikolay@molgen.mpg.de](mailto:nikolay@molgen.mpg.de)

SUPPLEMENTARY DATA

**
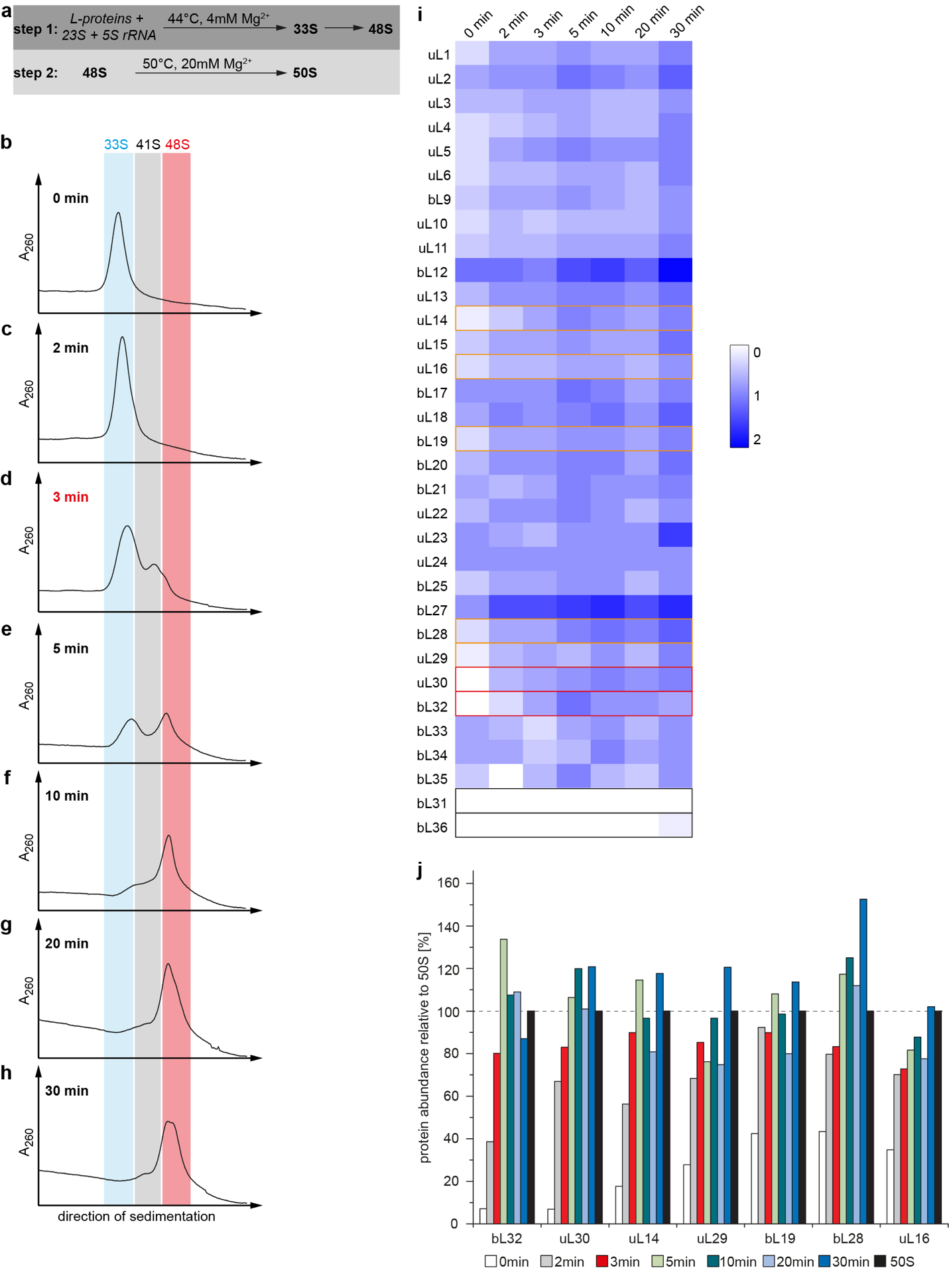
**

Supplementary Fig. 1: 50S *in vitro* reconstitution assay and subsequent analyses

**a)** Two step *in vitro* reconstitution assay of the 50S subunit. In step 1, 23S and 5S rRNA together with L-proteins incubate at 44°C for up to 30 min and are spontaneously converted into a 33S particle, which ultimately matures to 48S particles. During step 2, 48S particles (material in the red zone (b-h)) are quantitatively transformed into 50S like particles after 90 min at 50°C. **b-h)** step 1 time course reaction. Samples incubated under step 1 conditions as indicated were subjected to sucrose density gradient ultracentrifugation. **i, j)** quantitative mass spectrometry analysis of material incubated under step 1 conditions, subjected to sucrose cushion purification and LC-MS. Relative abundance of all L-proteins plotted as heat map in fold change **(i)** and selected L-proteins plotted as bar chart in % relative to 50S **(j)**. Experiments were performed in technical duplicates (n=2) and results represent mean values. Source data are provided as a Source Data file.


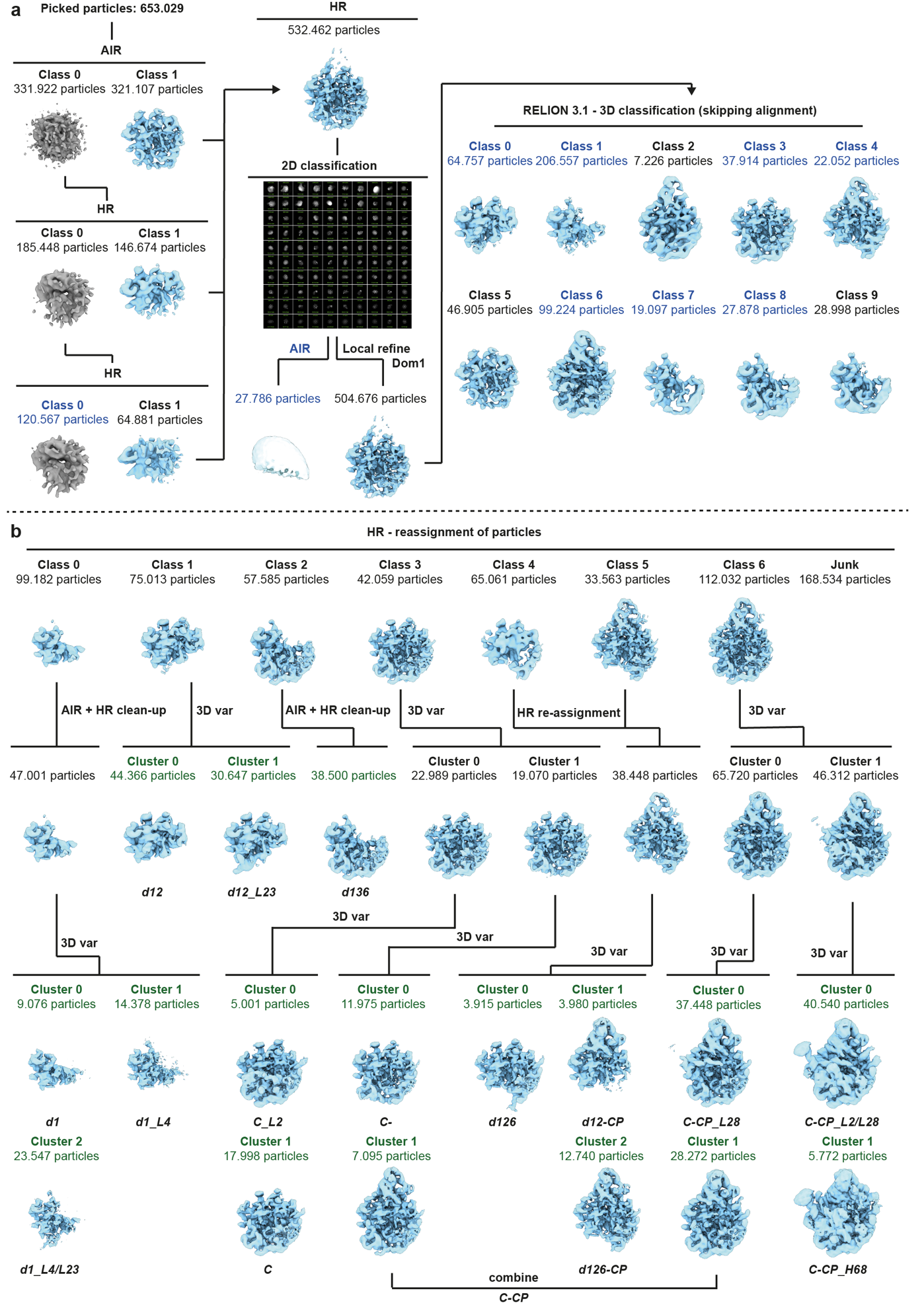


Supplementary Fig. 2: Sorting scheme

**a)** Sorting of particles and identification of initial classes. Extracted particles were subjected to an ab-initio reconstruction (AIR), followed by two rounds of heterogeneous refinement (HR) to recover ribosomal particles. Selected particles were refined to a consensus map, and remaining ice particles were sorted out using 2D classification. Particles were aligned to the 23S rRNA domain 1 region using local refinement and further sorted using 3D classification, skipping alignment in Relion 3.1. **b)** Re-assignment of initial classes, cleaning, and identification of final classes. Particles were re-assigned and re-aligned to previously identified classes. Templates used for particle re-assignment are labeled in blue. An additional bait template was generated using an AIR from selected non-ribosomal 2D classes. Further structural heterogeneity was identified using hierarchical 3D variability clustering. For *d1* and *d136* classes, assigned particles were cleaned using AIR followed by HR to identify the most defined subsets. Therefore, non-structured classes from AIR were used as bait templates, together with the starting ribosomal map in HR. Final classes are labeled in green. Sorting was performed at a pixel size of 3.75 Å. Final classes were refined at a pixel size of 1.25 Å (Supplementary Fig.3).


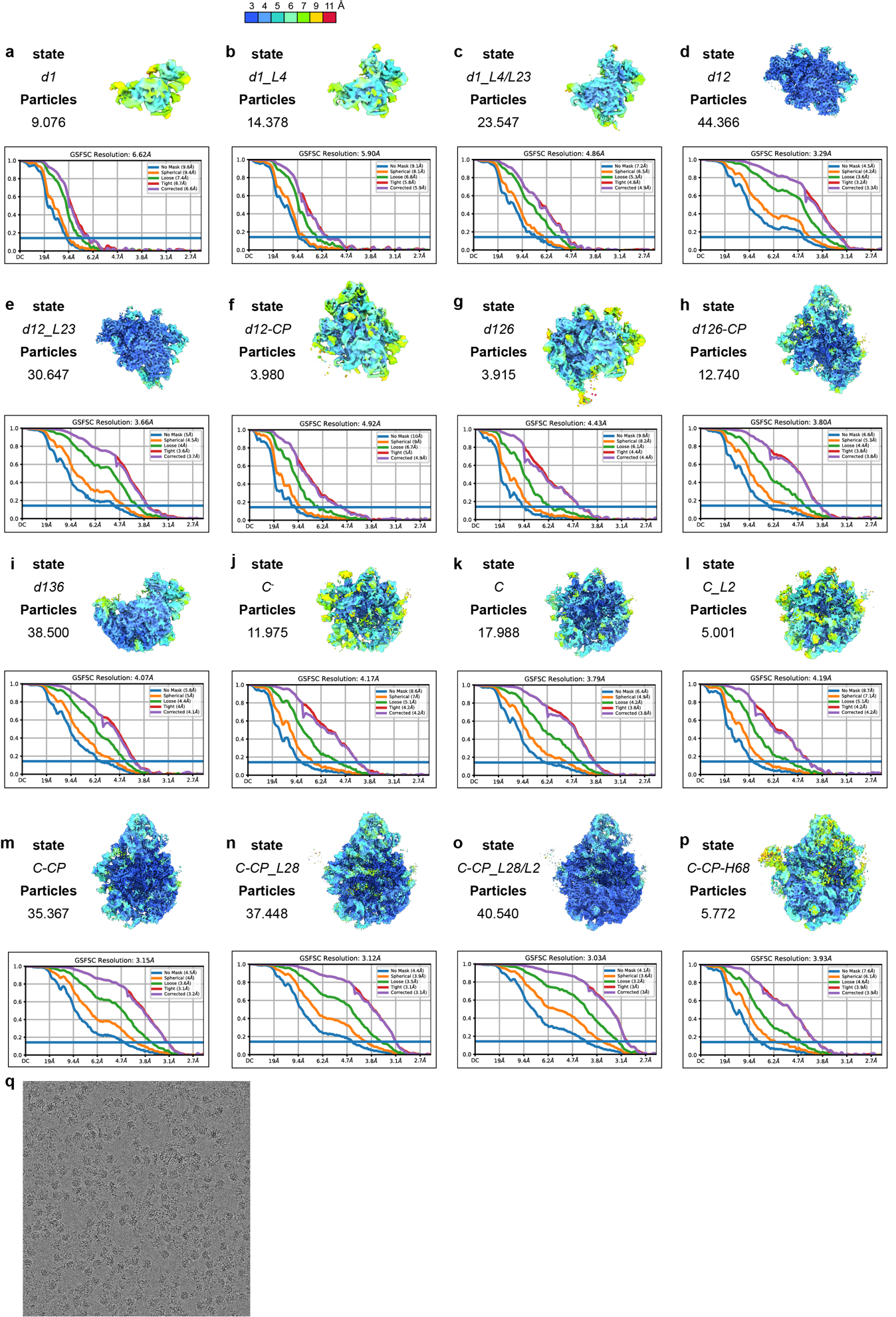


Supplementary Fig. 3: Final Cryo-EM maps, validation, and representative micrograph

Local resolution maps, total number of particles and gold-standard Fourier shell correlation (GSFSC) plots are shown for each state. States **a)** *d1*, **b)** *d1_L4*, **c)** *d1_L4/L23*, **d)** *d12*, **e)** *d12_L23*, **f)** *d12-CP*, **g)** *d126* and **i)** *d136* shown in back view, states **h)** *d126-CP*, **j)** *C^-^*, **k)** *C*, **l)** *C_L2*, **m)** *C-CP*, **n)** *C-CP_L28*, **o)** *C-CP_L28/L2* and **p)** *C-CP-H68* shown in crown view. Local resolution is shown using color keys from 3 Å (blue) to 11 Å (red). Overall resolutions were calculated from half-maps using the GSFC criteria cutoff of 0.143. **q)** Representative raw micrograph.
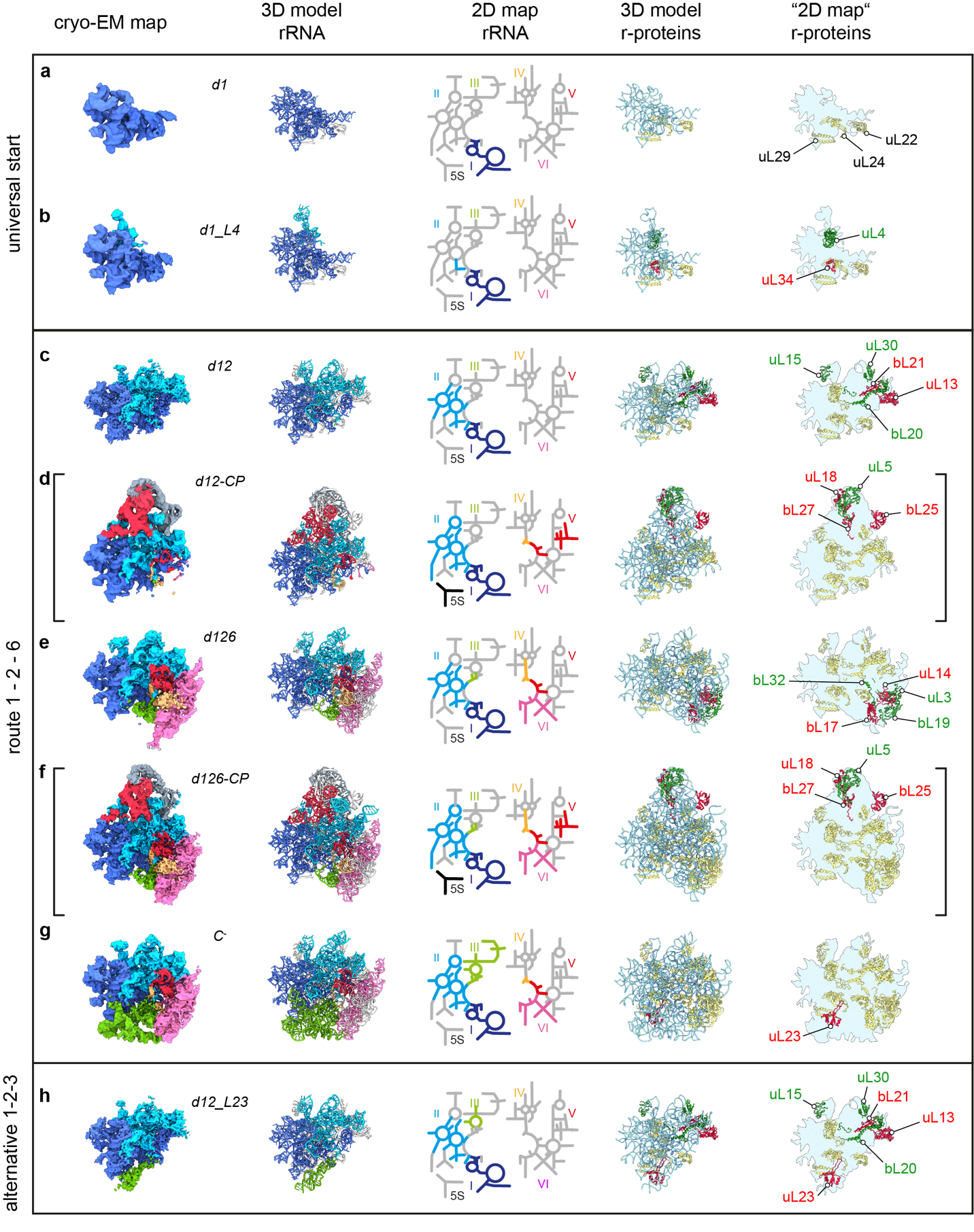


**
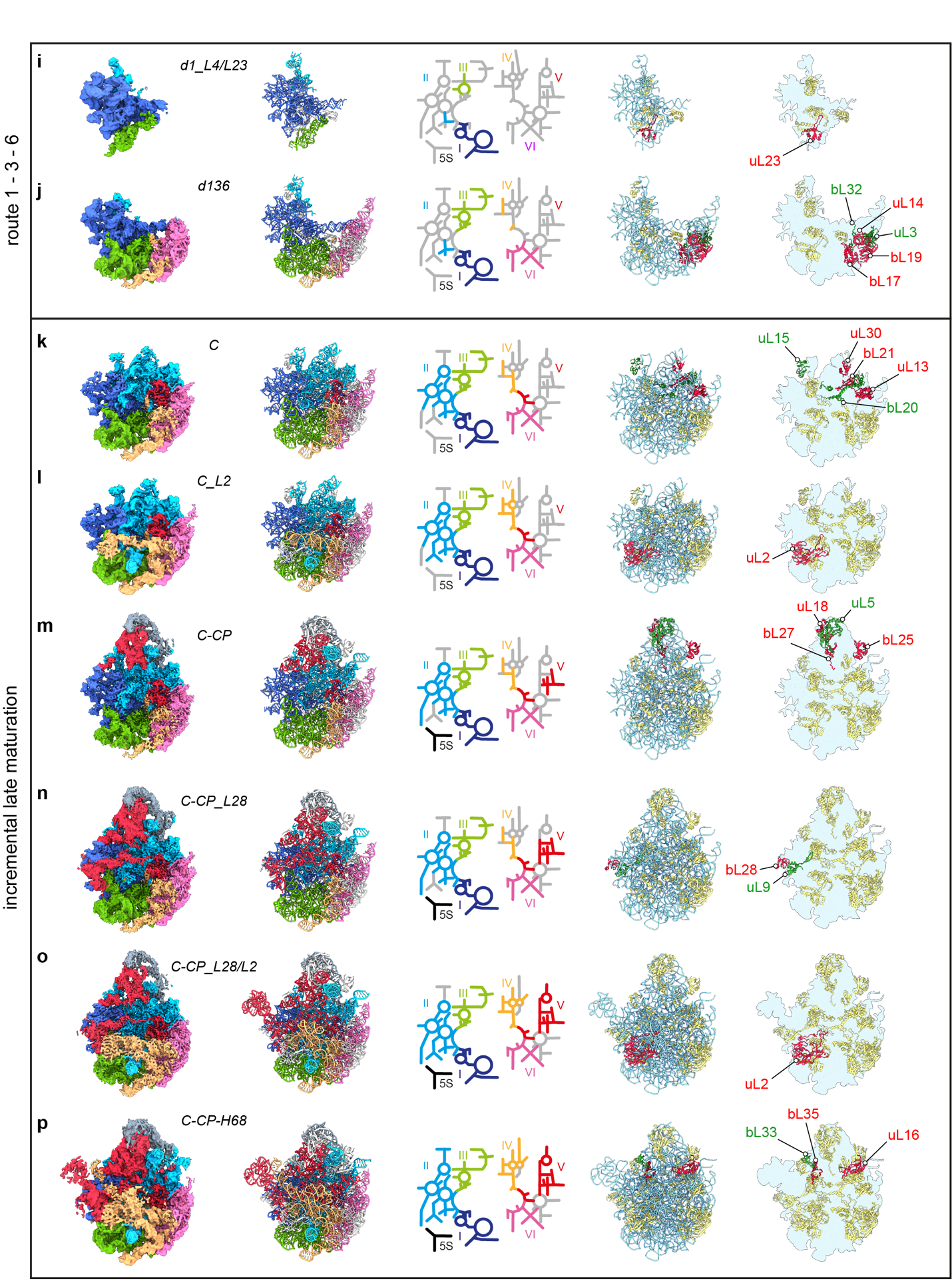
Supplementary Fig. 4: Gallery of pre-50S states**

**a-p)** States were lined up by their degree of structural completeness and their routes of maturation. Cryo-EM maps with rRNA and L-proteins color-coded according to the six architectural domains of the 23S rRNA they are part of. 3D PDB models, and 2D maps with the six rRNA domains color-coded. Colored regions indicate stably formed segments, for which cryo-EM densities were obtained. 3D PDB models, and “2D maps” with rRNA in pale blue and L-proteins in gold. Proteins for which density appears compared to the previous state are highlighted in red or green. **d and f)** Early assembly intermediates with cryo-EM density for the CP, *d12-CP* and *d126-CP*, respectively.


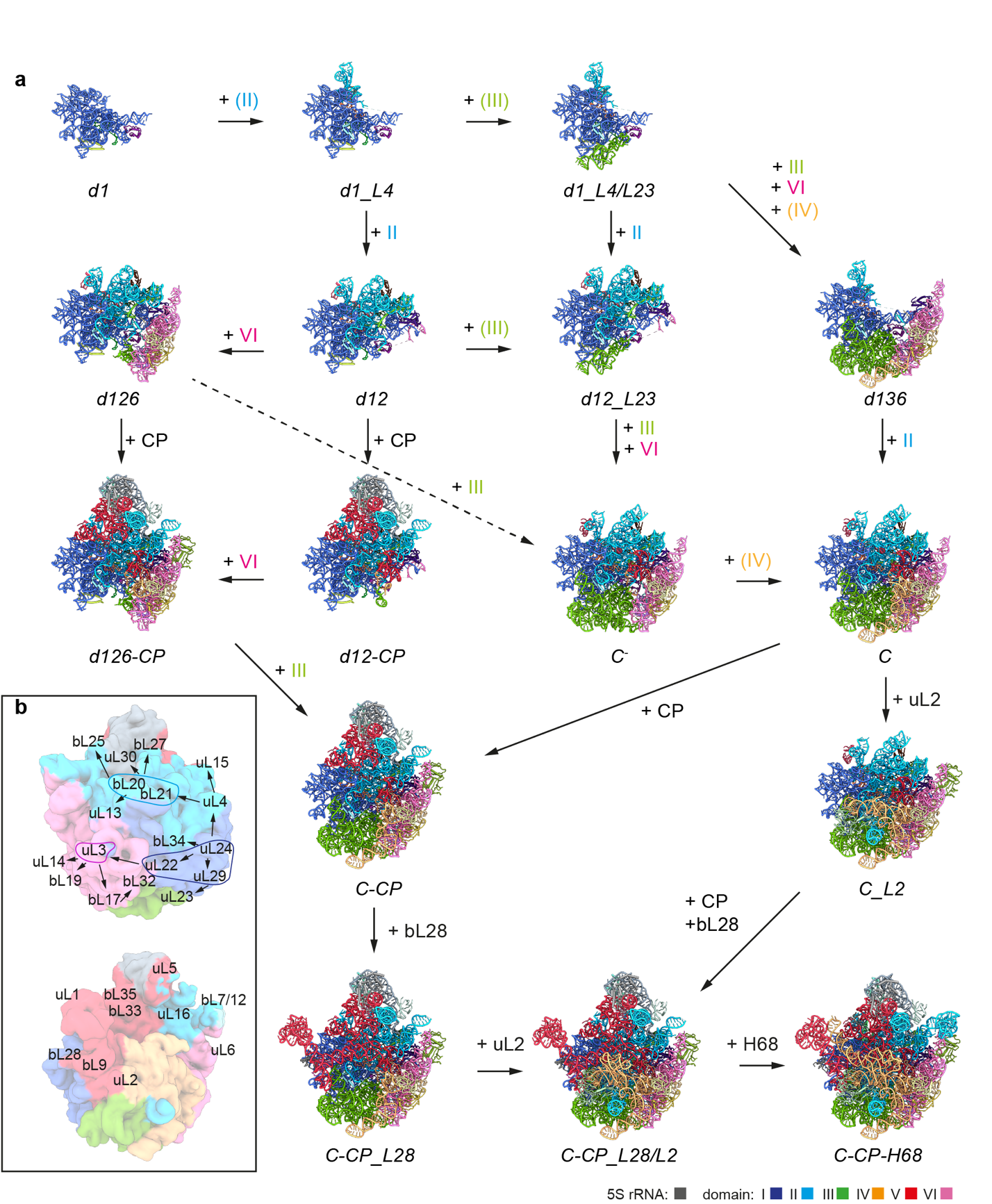


Supplementary Fig. 5: Interconversion of pre-50S states

**a)** PDB models of the individual states, with rRNA color-coded as specified. Sequences of appearing features (L-proteins or rRNA elements) are indicated. **b)** Possible order of L-protein binding, starting with uL24. The rRNA domains in brackets indicate partial formation of these domains.


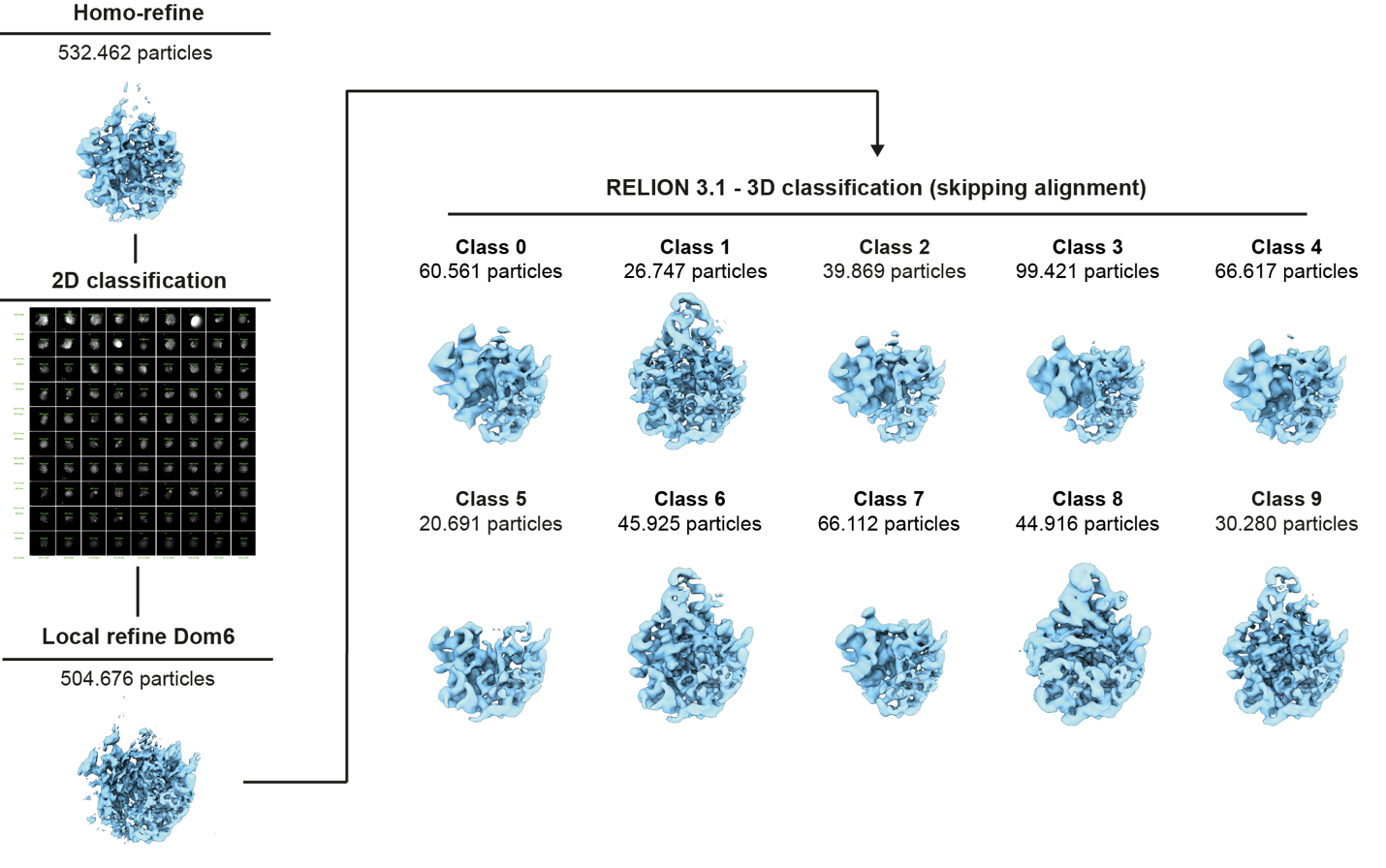


**Supplementary Fig. 6: Control sorting scheme**

Ribosomal particles from Supplementary Fig.2 were aligned to a consensus 50S map, cleaned by 2D classification and aligned to domain VI using local refinement. Subsequently, aligned particles were subjected to 3D classification skipping alignment in Relion 3.1.


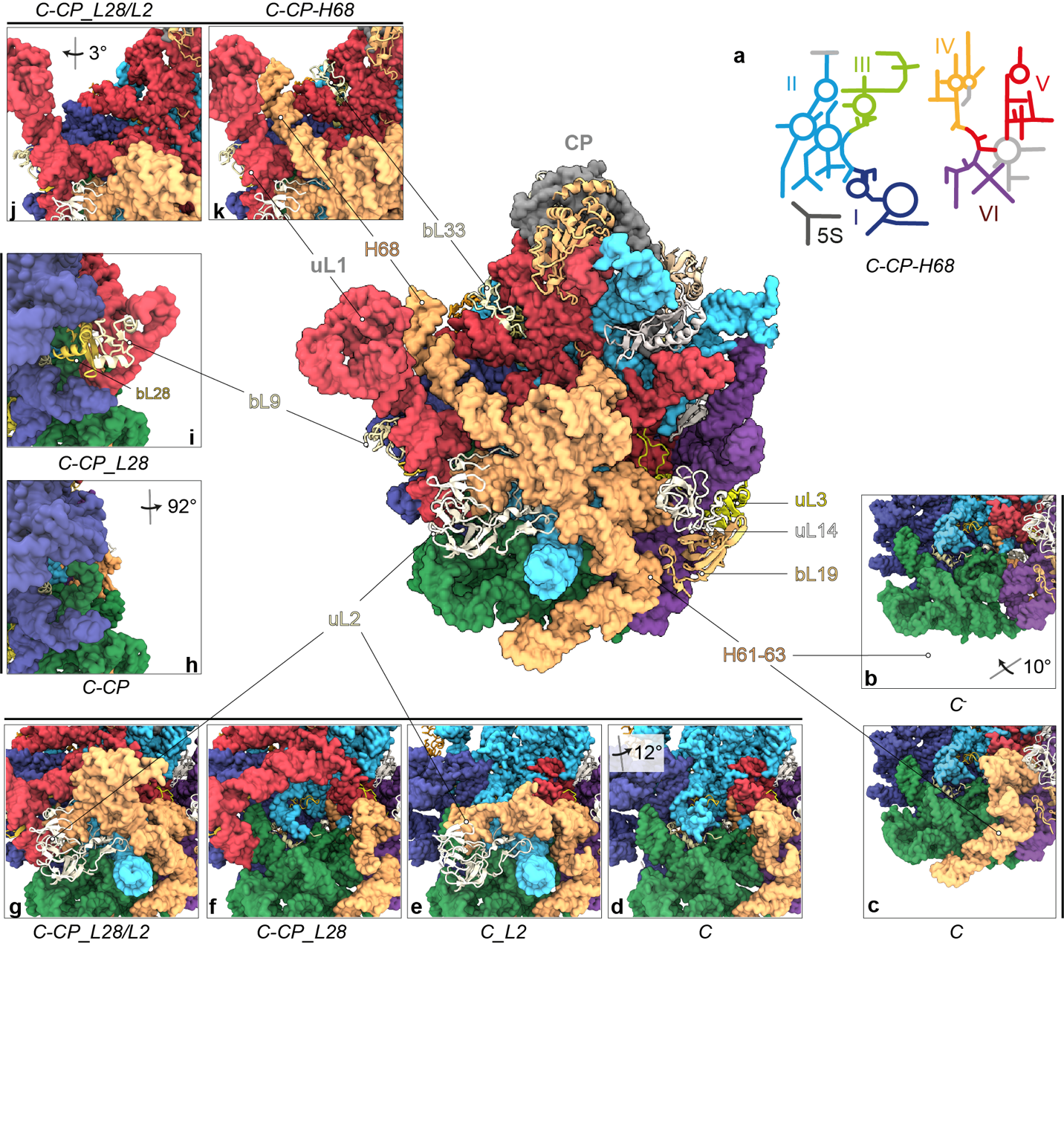


**Supplementary Fig. 7: Incremental late 50S assembly**

**a)** 2D rRNA map and atomic model of state *C-CP-H68* in crown view. L-proteins appear as cartoons, rRNA as surface model (lowpass-filtered to 5 Å resolution), color-coded as in the 2D-map. Lower sections of states *C^-^* **(b)** and *C* **(c)** lacking or exhibiting helices H61-63, respectively. **d-g)** Lower section of the individual states with absence or presence of uL2. Close-ups of states *C-CP* **(h)** and *C-CP_L28* **(i),** lacking or exhibiting bL28, bL9 and L1-stalk, respectively. Close-ups of states *C-CP_L28/L2* **(j)** and *C-CP-H68* **(k)** lacking or exhibiting bL33 and H68, respectively. CP, central protuberance. Viewing angles in b), d), h) and j) are shown relative to the full model of *C-CP-H68* (A).


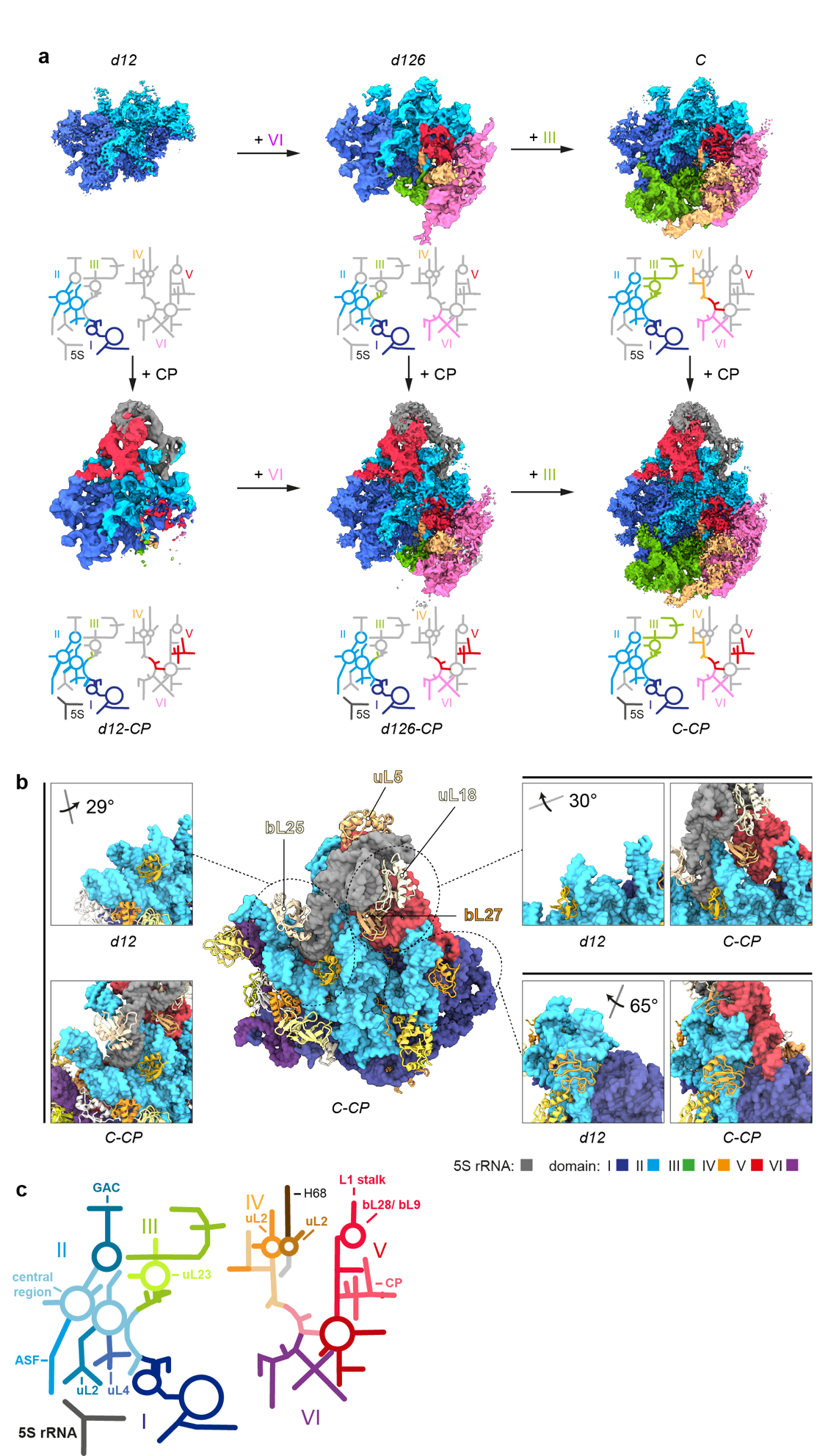


Supplementary Fig. 8: Docking of the 5S RNP

**a)** Cryo-EM maps of states *d12* (*-CP*), *d126* (*-CP*) and *C* (*-CP*), without and with cryo-EM density for the CP, dependent on docking of the 5S RNP, consisting of 5S rRNA, uL5 and uL18. **b)** Models of indicated states with rRNA as surface model (lowpass-filtered to 5 Å resolution), and L-proteins as cartoons. Selected regions are shown in detail, in absence and presence of the CP. Domains of the rRNA color-coded as indicated. Viewing angles shown relative to the full model (*C-CP*). **c)** 23S rRNA 2D map with the individual domains color-coded as indicated, binding sites of L-proteins and features of rRNA regions labeled. GAC, GTPase associated center (H42-44); CP, central protuberance (H80-88) and 5S rRNA; central region of domain II (pale blue); ASF, A-site finger (H38); L1 stalk (H 76-78).

|  | #1  *d1*  EMDB-16509  PDB 8C9C | #2  *d1_L4*  EMD16508  PDB 8C9B | #3  *d1_L4/L23*  EMD-16507 PDB 8C9A | #4  *d12*  EMD-16506 PDB 8C99 | #5 *d12_L23*  EMD-16505  PDB 8C98 | | #6  *d136*  EMD-16504  PDB 8C97 | #7  *d126*  EMD-16503  PDB 8C96 | #8  *d12-CP*  EMD-16502  PDB 8C95 |
| --- | --- | --- | --- | --- | --- | --- | --- | --- | --- |
| **Data collection and processing** |  |  |  |  |  |  | |  |  |
| Magnification | 31,000x | 31,000x | 31,000x | 31,000x | 31,000x | 31000x | | 31,000x | 31,000x |
| Voltage (kV) | 300 | 300 | 300 | 300 | 300 | 300 | | 300 | 300 |
| Electron exposure (e–/Å^2^) | 62 | 62 | 62 | 62 | 62 | 62 | | 62 | 62 |
| Defocus range (μm) | -0.5-2.0 | -0.5-2.0 | -0.5-2.0 | -0.5-2.0 | -0.5-2.0 | -0.5-2.0 | | -0.5-2.0 | -0.5-2.0 |
| Pixel size (Å) | 0.625  (1.25) | 0.625  (1.25) | 0.625 (1.25) | 0.625 (1.25) | 0.625 (1.25) | 0.625  (1.25) | | 0.625 (1.25) | 0.625 (1.25) |
| Symmetry imposed | C1 | C1 | C1 | C1 | C1 | C1 | | C1 | C1 |
| Initial particle images (no.) | 653,029 | 653,029 | 653,029 | 653,029 | 653,029 | 653,029 | | 653,029 | 653,029 |
| Final particle images (no.) | 9,076 | 14,378 | 23,547 | 44,366 | 30,647 | 38,500 | | 3,915 | 3,980 |
| Map resolution (Å) | 6.62 | 5.90 | 4.86 | 3.29 | 3.66 | 4.07 | | 4.43 | 4.92 |
| FSC threshold | 0.143 | 0.143 | 0.143 | 0.143 | 0.143 | 0.143 | | 0.143 | 0.143 |
| Map resolution range (Å) | 3.88-21.17 | 3.47-30 | 3.13-30 | 2.75-30 | 2.72-30 | 2.70-30 | | 2.68-30 | 2.96-30 |
|  |  |  |  |  |  |  | |  |  |
| **Refinement** |  |  |  |  |  |  | |  |  |
| Initial model used (PDB code) | 6GC7 | 6GC7 | 6GC7 | 6GC7 | 6GC7 | 6GC7 | | 6GC7 | 6GC7 |
| Model resolution (Å) | 6.5 | 5.8 | 4.8 | 3.3 | 3.6 | 4.0 | | 4.4 | 4.9 |
| FSC threshold | 0.143 | 0.143 | 0.143 | 0.143 | 0.143 | 0.143 | | 0.143 | 0.143 |
| Model resolution range (Å) | 6.2-8 | 5.6-7.3 | 4.6-5.7 | 3.2-3.5 | 3.6-3.9 | 3.9-4.5 | | 4.3-5.5 | 4.7-7 |
| Map sharpening *B* factor (Å^2^) | -272.1 | -218.6 | -150.9 | -70.9 | -73 | -96.5 | | -47.9 | -101.4 |
| Model composition  Non-hydrogen atoms | 13,032 | 15,782 | 18,061 | 27,988 | 30,289 | 36,169 | | 44,218 | 40,174 |
| Protein residues  Ligands | 256  0 | 441  0 | 525  0 | 987  0 | 1080  0 | 1077  0 | | 1607  0 | 1460  0 |
| *B* factors (Å^2^)  Protein  Ligand | -485  0 | -465  0 | -385  0 | -135  0 | -495  0 | -495  0 | | -315  0 | -440  0 |
| R.m.s. deviations  Bond lengths (Å)  Bond angles (°) | 0002 (0)  0.529 (3) | 0.002(0)  0.535 (0) | 0.002(0)  0.557(2) | 0.002(0)  0.535(0) | 0.002(0  0.522(1) | 0.002(0)  0.515(2) | | 0.002(0)  0.519(0) | 0.002(0)  0.560(7) |
| Validation  MolProbity score  Clashscore  Poor rotamers (%) | 1.87  8.6  0.47 | 1.65  7.72  0.27 | 1.6  6.58  0.23 | 1.61  5.35  0 | 1.64  5.79  0 | 1.66  5.93  0 | | 1.63  6.57  0.08 | 1.83  8.94  0.09 |
| Ramachandran plot  Favored (%)  Allowed (%)  Disallowed (%) | 93.95  6.05  0 | 96.49  3.51  0 | 96.45  3.55  0 | 95.31  4.69  0 | 95.33  4.67  0 | 95.22  4.58  0.19 | | 96.11  3.82  0.06 | 94.94  5.06  0 |

|  | #9  *d126-CP*  EMD-16501 PDB 8C94 | #10  *C^-^*  EMD-16500  PDB 8C93 | #11  *C*  EMD-16499  PDB 8C92 | #12  *C_L2*  EMD-16498  PDB 8C91 | #13  *C-CP*  EMD-16497  PDB 8C90 | #14  *C-CP_L28*  EMD-16496  PDB 8C8Z | | #15  *C-CP_L28/L2*  EMD-16495  PDB 8C8Y | #16  *C-CP-H68*  EMD-16494  PDB 8C8X |
| --- | --- | --- | --- | --- | --- | --- | --- | --- | --- |
| **Data collection and processing** |  |  |  |  |  | |  |  |  |
| Magnification | 31,000x | 31,000x | 31,000x | 31,000x | 31,000x | 31,000x | | 31,000x | 31,000x |
| Voltage (kV) | 300 | 300 | 300 | 300 | 300 | 300 | | 300 | 300 |
| Electron exposure (e–/Å^2^) | 62 | 62 | 62 | 62 | 62 | 62 | | 62 | 62 |
| Defocus range (μm) | -0.5-2.0 | -0.5-2.0 | -0.5-2.0 | -0.5-2.0 | -0.5-2.0 | -0.5-2.0 | | -0.5-2.0 | -0.5-2.0 |
| Pixel size (Å) | 0.625 (1.25) | 0.625  (1.25) | 0.625  (1.25) | 0.625  (1.25) | 0.625  (1.25) | 0.625  (1.25) | | 0.625  (1.25) | 0.625  (1.25) |
| Symmetry imposed | C1 | C1 | C1 | C1 | C1 | C1 | | C1 | C1 |
| Initial particle images (no.) | 653,029 | 653,029 | 653,029 | 653,029 | 653,029 | 653,029 | | 653,029 | 653,029 |
| Final particle images (no.) | 12,740 | 11,975 | 17,988 | 5,001 | 35,367 | 37,448 | | 40,540 | 5,772 |
| Map resolution (Å) | 3.80 | 4.17 | 3.79 | 4.19 | 3.15 | 3.12 | | 3.03 | 3.93 |
| FSC threshold | 0.143 | 0.143 | 0.143 | 0.143 | 0.143 | 0.143 | | 0.143 | 0.143 |
| Map resolution range (Å) | 2.70-30 | 2.68-30 | 2.70-30 | 2.68-30 | 2.75-30 | 2.75-30 | | 2.7-30 | 2.70-30 |
|  |  |  |  |  |  |  | |  |  |
| **Refinement** |  |  |  |  |  |  | |  |  |
| Initial model used (PDB code) | 6GC4 | 6GC7 | 6GC7 | 6GC6 | 6GC4 | 6GC4 | | 6GC4 | 6GBZ |
| Model resolution (Å) | 3.8 | 4.2 | 3.8 | 4.2 | 3.1 | 3.1 | | 3.0 | 3.9 |
| FSC threshold | 0.143 | 0.143 | 0.143 | 0.143 | 0.143 | 0.143 | | 0.143 | 0.143 |
| Model resolution range (Å) | 3.7-4.3 | 4.3-5.2 | 3.7-4.2 | 4.1-5.2 | 3.1-3.5 | 3.1-3.4 | | 3.0-3.2 | 3.9-4.6 |
| Map sharpening *B* factor (Å^2^) | -63.3 | -53.8 | -64.7 | -49.5 | -55.0 | -53.7 | | -54.6 | -48.0 |
| Model composition  Non-hydrogen atoms | 54,564 | 50,322 | 53,494 | 59,114 | 64,731 | 67,995 | | 77,322 | 82,783 |
| Protein residues  Ligands | 2,070  0 | 1,657  0 | 1,700  0 | 1,860  0 | 2,183  0 | 2,311  0 | | 2,566  0 | 2,832  0 |
| *B* factors (Å^2^)  Protein  Ligand | -165  0 | -290  0 | -175  0 | -250  0 | -135  0 | -110  0 | | -65  0 | -170  0 |
| R.m.s. deviations  Bond lengths (Å)  Bond angles (°) | 0002 (0)  0.563 (10) | 0.002(0  0.505 (0) | 0.002(0  0.511(0) | 0.002(0)  0.499(0) | 0.002(0  0.528(2) | 0.002(0)  0.556(14) | | 0.002(0)  0.536(4) | 0.004(10)  0.539(10) |
| Validation  MolProbity score  Clashscore  Poor rotamers (%) | 1.77  7.44  0 | 1.67  6.07  0.07 | 1.63  5.69  0.00 | 1.57  5.51  0.07 | 1.64  5.55  0.11 | 1.82  6.67  0.05 | | 1.67  4.85  0 | 1.69  6.35  0.3 |
| Ramachandran plot  Favored (%)  Allowed (%)  Disallowed (%) | 94.71  5.29  0 | 95.11  4.89  0 | 95.42  4.52  0.06 | 96.08  3.75  0.17 | 95.04  4.92  0.05 | 92.96  6.96  0.09 | | 93.7  6.22  0.08 | 95.09  4.83  0.07 |

**Supplementary Table 1:** **Cryo-EM data collection, refinement, and validation statistics**
