# Supplementary material for: Cryo-EM captures early ribosome assembly in action
Source: Nat Commun. 2023 Feb 17;14:898. doi: 10.1038/s41467-023-36607-9 (PMC9935924; doi:10.1038/s41467-023-36607-9)
Supplement: Supplementary file 3 — Description of Additional Supplementary Files [file 41467_2023_36607_MOESM3_ESM.pdf]

File name: Supplementary Movie 1

Description: Relative flexibility of rRNA domains. For each state, 10 serial Cryo-EM maps along an orthogonal variability trajectory were reconstructed, filtered to 8Å and shown as volume series, with the six rRNA domains colored according to the default code. Proteins transparent in gold and silver colors as indicated. Atomic models of proteins shown as ribbons in the same colors. While the domains I, III and VI are intrinsically rigid, they are flexible relative to each other, which enhances cooperativity. Once formed, the L1 stalk remains flexible, while the CP´s flexibility is terminated by incorporation of bL33, bL35 and uL2.

File name: Supplementary Movie 2

Description: Local seeding and cooperativity. Cryo-EM maps of *d1* states with rRNA domains according to the default color code. Proteins transparent in gold and silver colors as indicated. Atomic models of proteins shown as ribbons in the same colors. Minimal set of L-proteins (uL24, uL22 and uL29) in state *d1* and uL4 and uL23 mediated seeding of adjacent rRNA helices.

File name: Supplementary Movie 3

Description: Maturation of the subunit´s core. Cryo-EM maps of *d1* states with rRNA domains according to the default color code. Proteins transparent in gold and silver colors as indicated. Atomic models of proteins shown as ribbons in the same colors. Minimal set of L-proteins (uL24, uL22 and uL29) in state *d1* and uL4 and uL23 mediated seeding of adjacent rRNA helices.
