# Supplementary material for: Cryo-EM captures early ribosome assembly in action
Source: Nat Commun. 2023 Feb 17;14:898. doi: 10.1038/s41467-023-36607-9 (PMC9935924; doi:10.1038/s41467-023-36607-9)
Supplement: Supplementary file 7 — Reporting Summary [file 41467_2023_36607_MOESM7_ESM.pdf]

nature portfolio

Corresponding author(s): Christian M.T.Spahn
Rainer Nikolay

Last updated by author(s): 2023/01/27

Reporting Summary

Nature Portfolio wishes to improve the reproducibility of the work that we publish. This form provides structure for consistency and transparency in reporting. For further information on Nature Portfolio policies, see our Editorial Policies and the Editorial Policy Checklist.

Statistics

For all statistical analyses, confirm that the following items are present in the figure legend, table legend, main text, or Methods section.

n/a	Confirmed	
☐	☒	The exact sample size (*n*) for each experimental group/condition, given as a discrete number and unit of measurement
☐	☒	A statement on whether measurements were taken from distinct samples or whether the same sample was measured repeatedly
☒	☐	The statistical test(s) used AND whether they are one- or two-sided<br>*Only common tests should be described solely by name; describe more complex techniques in the Methods section.*
☒	☐	A description of all covariates tested
☒	☐	A description of any assumptions or corrections, such as tests of normality and adjustment for multiple comparisons
☐	☒	A full description of the statistical parameters including central tendency (e.g. means) or other basic estimates (e.g. regression coefficient) AND variation (e.g. standard deviation) or associated estimates of uncertainty (e.g. confidence intervals)
☒	☐	For null hypothesis testing, the test statistic (e.g. *F*, *t*, *r*) with confidence intervals, effect sizes, degrees of freedom and *P* value noted<br>*Give P values as exact values whenever suitable.*
☒	☐	For Bayesian analysis, information on the choice of priors and Markov chain Monte Carlo settings
☒	☐	For hierarchical and complex designs, identification of the appropriate level for tests and full reporting of outcomes
☒	☐	Estimates of effect sizes (e.g. Cohen's *d*, Pearson's *r*), indicating how they were calculated

Our web collection on statistics for biologists contains articles on many of the points above.

Software and code

Policy information about availability of computer code

Data collection	Cryo-electron microscopy data collection utilized the following software/code: EPU (FEI company), Leginon (Carragher et al., 2000; Suloway et al., 2005)
Data analysis	Cryo-electron microscopy data analysis utilized the following software/code: Cryosparc v3.1 (Punjani et al., 2017), COOT 0.9.4 (Emsley and Cowtan, 2004), ERRASER (Chou et al., 2013), Gautomatch (developed by k. Zhang), Gctf (Zhang, 2016), MDFit (Whitford et al., 2011), MotionCor2 (Zheng et al., 2017), PHENIX 1.19 (Adams et al., 2010), Relion 3.0 and Relion 3.1 (Zivanov et al., 2018), Spider (Frank et al.1996), UCSF Chimera v1.10.2 (Pettersen et al., 2004), UCSF ChimeraX 1.3 (Goddard et al., 2018), Xmipp3 2D classification (de la Rosa-Trevín et al., 2013). Statistical analysis utilized the following software/code: Heatmap Illustrator 1.0 (http://hemi.biocuckoo.org/down.php) and Origin8 (https://www.originlab.com/).

For manuscripts utilizing custom algorithms or software that are central to the research but not yet described in published literature, software must be made available to editors and reviewers. We strongly encourage code deposition in a community repository (e.g. GitHub). See the Nature Portfolio guidelines for submitting code & software for further information.

Data

Policy information about availability of data

All manuscripts must include a data availability statement. This statement should provide the following information, where applicable:

- Accession codes, unique identifiers, or web links for publicly available datasets
- A description of any restrictions on data availability
- For clinical datasets or third party data, please ensure that the statement adheres to our policy

1) d1:d1: PDB ID 8C9C, EMD-16509

2) d1_L4: PDB ID 8C9B, EMD-16508

3) 1_L4/L23: PDB ID 8C9A, EMD-16507

4) d12: PDB ID 8C99, EMD-16506

5) d12_L23: PDB ID 8C98, EMD-16505

6) d136: PDB ID 8C97, EMD-16504

7) d126: PDB ID 8C96, EMD-16503

8) d12-CP: PDB ID 8C95, EMD-16502

9) d126-CP: PDB ID 8C94, EMD-16501

10) C-: PDB ID 8C93, EMD-16500

11) C: PDB ID 8C92, EMD-16499

12) C_L2: PDB ID 8C91, EMD-16498

13) C-CP: PDB ID 8C90, EMD-16497

14) C-CP_L28: PDB ID 8C8Z, EMD-16496

15) C-CP_L28/L2: PDB ID 8C8Y, EMD-16495

16) C-CP-H68: PDB ID 8C8X, EMD-16494

Mass spectrometry proteomics data have been deposited to ProteomeXchange Consortium (http://proteomecentral.proteomexchange.org) via the PRIDE partner repository with the dataset identifier PXD030312.

Human research participants

Policy information about studies involving human research participants and Sex and Gender in Research.

Reporting on sex and gender	N/A
Population characteristics	N/A
Recruitment	N/A
Ethics oversight	N/A

Note that full information on the approval of the study protocol must also be provided in the manuscript.

Field-specific reporting

Please select the one below that is the best fit for your research. If you are not sure, read the appropriate sections before making your selection.

☒ Life sciences ☐ Behavioural & social sciences ☐ Ecological, evolutionary & environmental sciences

For a reference copy of the document with all sections, see nature.com/documents/nr-reporting-summary-flat.pdf

Life sciences study design

All studies must disclose on these points even when the disclosure is negative.

Sample size	For the cryo-EM analysis the number of particle images was limited by the available beam time.
Data exclusions	Cryo-EM reconstructions corresponding to contaminations (ice, rRNA etc.) were removed from the analysis, as described in the sorting scheme (Supplementary Fig.2).
Replication	Translation activity assays and quantitative mass spectrometry experiments were performed in duplicates. Sucrose density gradients and cryo-EM measurements were conducted once.
Randomization	Not relevant for this study.
Blinding	Not relevant for this study.

Reporting for specific materials, systems and methods

We require information from authors about some types of materials, experimental systems and methods used in many studies. Here, indicate whether each material, system or method listed is relevant to your study. If you are not sure if a list item applies to your research, read the appropriate section before selecting a response.

Materials & experimental systems

n/a	Involved in the study	
☒	☐	Antibodies
☒	☐	Eukaryotic cell lines
☒	☐	Palaeontology and archaeology
☒	☐	Animals and other organisms
☒	☐	Clinical data
☒	☐	Dual use research of concern

Methods

n/a	Involved in the study	
☒	☐	ChIP-seq
☒	☐	Flow cytometry
☒	☐	MRI-based neuroimaging
